# Are need for affect and cognition culture dependent? Implications for global public health campaigns: a cross-sectional study

**DOI:** 10.1186/s12889-021-10689-w

**Published:** 2021-04-09

**Authors:** Min Zhang, Bei Zhu, Chunlan Yuan, Chao Zhao, Jiaofeng Wang, Qingwei Ruan, Chao Han, Zhijun Bao, Jie Chen, Kevin ( Vin) Arceneaux, Ryan Vander Wielen, Greg J. Siegle

**Affiliations:** 1 Shanghai Key Laboratory of Clinical Geriatric Medicine, Huadong Hospital, Fudan University, 221 West Yan’an Road, Shanghai, China; 2 Jiuting Community Health Service Center, Shanghai, Songjiang District China; 3 National Clinical Research Center for Aging and Medicine, Huashan Hospital, Fudan University, Shanghai, China; 4 Department of Political Science, Temple University, Philadelphia, PA USA; 5 Department of Psychiatry, University of Pittsburgh, Pittsburgh, PA USA

**Keywords:** Intrinsic motivation, Need for affect, Need for cognition, Cultural differences, Global public health campaign

## Abstract

**Background:**

Cultural differences in affective and cognitive intrinsic motivation could pose challenges for global public health campaigns, which use cognitive or affective goals to evoke desired attitudes and proactive health-promoting actions. This study aimed to identify cross-cultural differences in affective and cognitive intrinsic motivation and discuss the potential value of this information for public health promotion.

**Methods:**

A cross-sectional survey using cross-culturally validated need for affect (NFA) and need for cognition (NFC) scales was carried out among 1166 Chinese participants, and the results were compared with published data from 980 American participants. Additionally, we assessed a highly prevalent symbolic geriatric health condition, hearing loss, in 500 Chinese community-dwelling seniors. The Chinese NFA scale was developed following the translation-back translation procedure, and the psychometric evaluation was performed by applying confirmatory factor analysis (CFA), exploratory structural equation modeling (ESEM), correlation analysis and multigroup invariance test. MANOVA and Hedge’s g statistic were employed to compare the NFA and NFC levels between individuals from different countries and between Chinese seniors with and without hearing loss. The relation of early hearing intervention intention to NFA and NFC was also explored in the Chinese sample.

**Results:**

A basic two-factor model of NFA adequately fit the sample data from Chinese and American cultures. The questionnaire demonstrated reasonable invariance of the factor structure and factor loadings across the groups. Those in the primary Chinese sample had lower NFA and NFC than their American peers. This difference held in the senior sample. Moreover, Chinese seniors with hearing loss had even lower NFA and NFC than those without hearing loss. Their early hearing intervention intention was low but was associated with intrinsic motivation.

**Conclusions:**

The Need for Affect (NFA) construct may be generalized beyond its Western origins. There was a general lack of affective and cognitive intrinsic motivation in Chinese individuals, particularly in seniors with hearing loss, compared with their American peers. These differences point to a potential challenge in framing effective messages for some cultures in the geriatric public health domain. Ideally, recognizing and understanding this challenge will inspire the consideration of novel persuasive strategies for these audiences.

**Supplementary Information:**

The online version contains supplementary material available at 10.1186/s12889-021-10689-w.

## Background

Many disciplines rely on persuasive communication with target audiences around affective goals (e.g., “do X to be happier”) or cognitive goals (e.g., “you will think more clearly”). An implicit assumption is that such affective goals are universal. Rather, affective and cognitive orientation (indexed by need for affect and need for cognition) differs across individuals and is associated with the effectiveness of messages in persuasive communication [1, 2] and consequent proactive behaviors [3]. Aquino et al. [4] explained the neural basis of this association by observing that the ventromedial prefrontal cortex (vMPFC) was specifically responsive when message receivers evaluated the match between a message and their affective and cognitive orientations. Affective and cognitive orientation may be particularly important for global persuasive communication efforts, e.g., public health campaigns, as similar campaign content might yield diverse outcomes across national cultures if there are cross-cultural differences in affective and cognitive orientation. In a review on structural matching effects in persuasive communication, Teeny et al. [5] pointed out that tailoring messages to cultural characteristics is a high-level matching strategy for persuasion enhancement.

Affective and cognitive orientations are indexed by self-report questionnaires. The Need for Affect (NFA) questionnaire assesses the tendency to approach or avoid emotion-inducing situations and activities. It focuses on the motivation to engage in the affective process rather than assessing emotional ability or emotional style [6]. People high in NFA tend to rely upon emotional information in attitude formation and the regulation of behavior [1, 2]. The Need for Cognition (NFC) questionnaire assesses the tendency to engage in and enjoy effortful thinking [7] and is related to rational decision-making [8] and effort expenditure [9]. Higher levels of NFA are associated with greater persuasion in response to an affect-based (but not cognition-based) persuasive message, whereas higher levels of NFC are associated with greater persuasion in response to a cognition-based (but not affect-based) persuasive message [1].

Geriatric health campaign messages are often geared towards affective goals (e.g., happiness, enjoyment), as evidence has shown that affective outcome expectancies have a significantly more positive impact on older people’s intentions to take proactive actions than rationale outcome expectancies [10]. However, cultural context may play a role in structuring affective goals. There are important differences across cultures in terms of the norms governing human engagement with emotions [11]. From the approach perspective of emotional engagement, East Asians – especially Chinese – differ from their Western counterparts in affective disposition [12]. For example, a unique study suggested that U.S. residents of Chinese descent tended to value high-arousal positive affect (e.g., excitement) less than Americans of specifically European descent [13]; they tended to want to maximize positive and minimize negative affect less than did Americans of European descent and therefore were more likely to feel the bad with the good, referred to as mixed affective experience [14]. From the avoidance perspective of emotional engagement, Chinese individuals are more affected by the perceived risk of potential losses than individuals from the Netherlands and the USA [15]. Compared to Americans, East Asians are less inclined to overestimate the emotional consequences of future events [16]. Additionally, there are differences across cultures in people’s engagement in thinking that might influence cognitive goal structures. The Confucian, Taoist and Buddhist philosophic traditions oppose public argumentation and debate [17] and focus on learning in a mechanical way without thought or meaning, which has evolved to the extent that people in such cultures are considered to lack abstract and critical thinking ability, to overemphasize concrete examples, and to lack originality and creativity [18].

Overall, these data suggest that the baseline levels of affective and cognitive intrinsic motivation may be low in certain cultures, so conventional emotion/cognition-based messaging is likely to be less effective. A prototypical example of diverse outcomes across cultures, given similar campaign content and service accessibility, is in the hearing healthcare domain. There are cross-cultural public health outreach efforts spanning China and the U.S. The prevalence of hearing loss in elderly individuals is 45 ~ 63% in both countries [19]. The WHO proposed a World Hearing Day (observed annually on March 3rd) to facilitate global hearing health campaigns. The themes of this day initially focused on the cognitive side, e.g., “*Make listening safe: avoid noise-induced hearing loss”, “Earcare can avoid hearing loss”, “Proper use of hearing aids”,* and *“Act now, here is how”*, while the more recent themes have emphasized the emotional side, e.g., “*Hearing for life: don’t let hearing loss limit you*”*, “Hear the future”, “Make a sound investment”,* and *“Healthy hearing, happy life”.* Although advocacy of early hearing intervention to reduce the long-term adverse effects of hearing loss has been carried out for over 2 decades using the same WHO messages, the hearing intervention rate in geriatrics remains low at < 2% in China [20], whereas it is approximately 16% in the U.S. [21]. Potentially, understanding differences in audiences’ affective and cognitive intrinsic motivation at a national level could help health professionals tailor persuasive messages to particular populations and hence facilitate successful campaigns globally.

The current study examines whether NFA and NFC differ systematically across relatively large Chinese and American samples. The objectives are to 1) develop a Chinese NFA scale based on Appel et al.’s English language version [22] and establish the validity of this scale; 2) examine the differences in NFA and NFC between our Chinese and American general public samples and perform a comparison with the European samples from Appel et al.’s study; and 3) taking hearing loss as an example, explore whether community-based seniors with a public health condition in the Chinese sample have reasonably high NFA or NFC, as assumed by public health campaigns, and examine whether their early hearing intervention intention is related to NFA and NFC. We hypothesized that the Chinese sample would have different levels of NFA and NFC than the American sample. We also hypothesized that Chinese seniors with hearing loss would be similar to those with normal hearing in terms of NFA and NFC, as no evidence has shown that hearing loss alters individuals’ affective and cognitive intrinsic motivation. We expected to observe a correlation between early hearing intervention and NFA and/or NFC in Chinese seniors, given that the Chinese hearing health campaign messages consist of both affective-driven and cognitive-driven content.

## Methods

### Participants

We recruited 1195 Chinese adults to participate in the study from April to July 2019. This sample size was comparable to those in the original NFA scale development studies [6, 22]. Of the 1195 participants (*age* = 45.4 ± 17.6 yrs., *range* = 20 ~ 90 yrs., 65.6% female), 695 (*age* = 30.3 ± 13.3 yrs., *range* = 20 ~ 78 yrs., 65.7% female) were recruited through an online survey tool (TengXun Survey) and completed a web-based survey in return for an instant and direct deposit of RMB¥2 RMB (USD$0.3) to their WeChat payment account. Five hundred older participants (*age* = 65.7 ± 10.5 yrs., *range* = 44 ~ 90 yrs., 64.6% female) were recruited offline at a local community health center during their annual mandatory physical examination. The community-based participants completed hearing assessments in addition to the NFA and NFC surveys. They were reimbursed ¥20 RMB for their participation and travel. Our American sample (*age* = 49.43 ± 13.8 yrs., *range* = 19 ~ 88 yrs., 68.0% female) was from Arceneaux and Vander Wielen [23]. The 1006 individuals in their study completed a web-based survey through Qualtrics in return for “online currency” that can be redeemed for consumer products or cash. Twenty-nine Chinese participants and twenty-six American participants had the same values across the NFA items and were suspected “straight-lining” cases lacking engagement and reporting reliability. Therefore, their data were excluded from the primary analyses.

### Instruments

#### English need for affect scale

The English 10-item short version consists of approach and avoidance subscales with 5 items in each [22]. The scale uses a 7-point Likert scale for responses [− 3 = Strongly disagree to 3 = Strongly agree, with 0 = Uncertain]. Some subsequent studies, including Arceneux and Vander Wielen [23], used a 5-point Likert scale for responses (1 = Strongly disagree, 5 = Strongly agree). The mean score of the items was used as the overall score of the NFA scale/subscales, in line with Appel et al. [22]

#### Chinese NFA scale construction

The Chinese version of the NFA (see Appendix) was rigorously developed in line with state-of-the-art standards in cross-cultural research using the translation-back translation method. The English NFA was translated into Chinese by the first author with assistance from a faculty member in the Chinese language department of Fudan University. The initial translation was tested on two bilingual doctoral students for content equivalence. For some items, the direct translation and standard back-translation procedures may not have captured equivalent emotion concepts in Chinese, as addressed in the literature [24, 25]. Discrepancies were discussed and resolved by consensus, and modifications to the translation were made as needed. The response scale was a 7-point Likert scale (− 3 = Strongly disagree to 3 = Strongly agree, with 0 = Uncertain). The final version was piloted in a sample of 3 undergraduate volunteers and 5 elderly volunteers. The volunteers indicated that the instructions and the items were easy to understand.

#### Need for cognition scale

Cacioppo et al.’s [7] 18-item scale was used to assess the NFC in an American sample. The Chinese NFC scale validated by Kuang et al. [26] was used in the Chinese sample. The Chinese NFC scale has an internal consistency of 0.89, a split reliability of 0.90, and a test-retest reliability of 0.86. It demonstrated a sound convergent validity in terms of a strong relationship with self-efficacy (*r* = 0.52, *p* < 0.01), which was in line with Cacioppo et al. [7]. The sum score was computed to index the overall score of the NFC scale, as suggested by Cacioppo et al. [7].

#### Hearing assessment

The purpose of assessing hearing impairment in the Chinese sample was to make claims regarding the extent to which, in Chinese culture, low levels of NFA are specifically present in the subpopulation that would be targeted by hearing campaigns. Two hearing-related assessments were administered to the community-based seniors: 1) a simplified pure tone examination [27] using an audiometer (Interacoustics, MA52) and the 10-item Hearing Handicap Inventory for the Elderly-Screening Questionnaire [28], which were used to estimate hearing loss severity; 2) a multiple choice intention question, namely, “When do you think you will consider wearing hearing aids and take serious action?” The five choices were: ①When/if I have mild hearing loss; ②When/if I have moderate hearing loss; ③When/if I have severe hearing loss and have difficulty communicating with others; ④When/if I lose all my hearing; and ⑤I will never consider wearing hearing aids because they are not helpful. This multiple-choice question was asked to measure an individual’s early hearing intervention intention, quantified by how early a proactive action might take place once the hearing problem is recognized, with the highest intention score being assigned to choice ① and the lowest intention score to choice ⑤.

#### Procedures

Chinese participants responded to the Chinese NFA questionnaire developed in the present study (on a 7-point Likert scale) and the Chinese NFC scale. The 500 community-based Chinese seniors completed the hearing assessments as well. Forty-seven of the 500 participants completed the battery twice with an interval of 3 ~ 4 weeks; on each occasion, they completed the NFA, NFC, and the single question about early hearing intervention intention. American participants in Arceneaux and Vander Wielen’s [23] study responded to the English NFA and NFC questionnaires.

#### Analysis methods

**Fig. 1:**
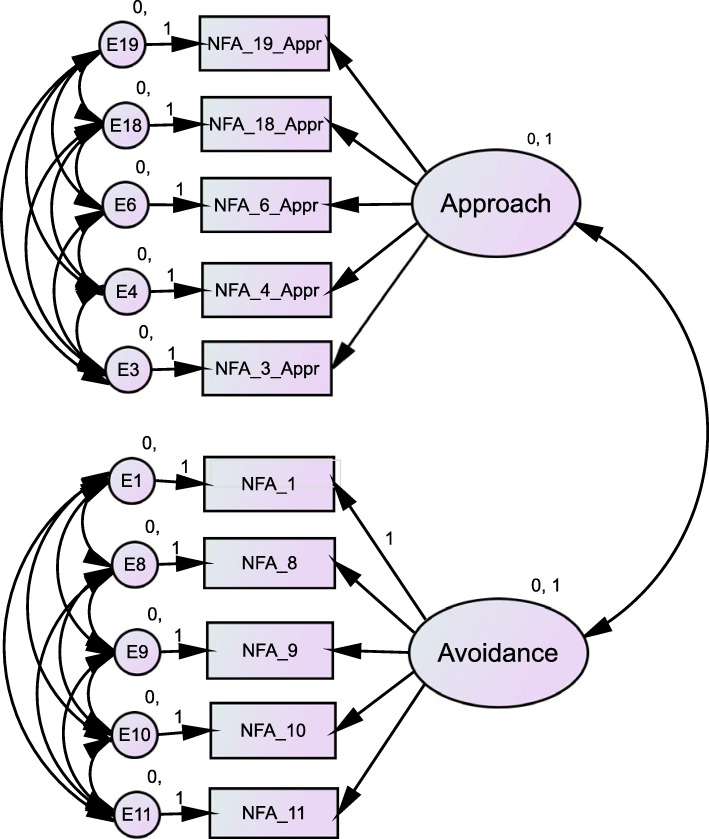
The one-level two-factor model of the 10-item Need for Affect scale. Items within each latent factor were allowed to correlate. Cross-loading was constrained to zero

Only 5.9% of cases had missing data; overall, therefore, 0.25% of the values were missing in our samples. Missing values were managed via a full information maximum likelihood (FIML) approach. The psychometrics of the Chinese NFA questionnaire were examined in terms of test-retest reliability, internal consistency and Cronbach’s alpha. Convergent validity was indexed by the correlation between the NFA and NFC scales [22]. The construct validity of the NFA scale in the Chinese and American samples was examined by testing the one-level two-factor model (Fig. 1) via confirmatory factor analysis and exploratory structural equation modeling (ESEM) with AMOS 24.0. We split our samples into 4 subsamples (2 Chinese subsamples and 2 American subsamples) (Table 1) in a random manner for the purposes of internal verification and replication of the model fit testing. Splitting samples in CFA is recommended by Fokkema and Greiff [29] to minimize model overfit. Various cutoff levels for the model fit indices have been recommended in previous literature [30]. Recent studies have suggested that in order to be meaningful, the thresholds should be considered in combination with important parameters (e.g., sample size and standardized factor loadings) [31–33].

**Table 1:** Demographics of the 4 subsamples split for the purpose of internal verification and replication of the model fit testing

	Chinese sample (1166) Subsample1 (581) Subsample2 (585)		American sample (980) Subsample1 (507) Subsample2 (473)	
**Age range (years)**	18–90	17–87	20–84	19–88
**Age mean (SD)**	45.62 (21.55)	45.29 (21.95)	48.86 (14.32)	50.04 (13.21)
**Female (%)**	63.3%	67.2%	69.0%	66.9%

In the present study, the following cut-off values were adopted: 1) comparative fit index (CFI), comparative fit index (TLI), and Turker and Lewis’s Index of fit (NFI) of 0.90 [34, 35]; 2) root-mean-square error of approximation (RMSEA) of 0.10 [36]. These criteria have been used by other researchers [37–39]. The goodness-of-fit results of the ESEM, a modified CFA model (allowing cross-loadings) and a basic CFA model (no cross-loadings) were reported. Although models with cross-loadings generally fit to a stronger degree, we were particularly interested in whether a basic CFA model could adequately fit for the reason that, the basic CFA model not only maximizes the theoretical interpretability of the NFA scale, it also matches the unit-weighted scoring approach applied in the NFA literature and generally preferred both in practical use and research on comparisons across multiple samples [40].

The configural, construct-level metric and scalar invariance of the basic NFA model between the Chinese and American samples were tested following a stepwise procedure [41, 42]. Specifically, a series of nested restrictive models were established and compared based on the CFI change (ΔCFI) and RMSEA change (ΔRMSEA) significance tests (see Table 4). Ideally, the invariance test could be conducted among the 4 subsamples, however, due to the lack of a clear cut-off between Chen’s (2007) [43] invariance criterion for 2-group condition and Rutkowski and Svetina’s (2014) [44] liberal criterion for 10- and 20-group condition, our invariance test was performed between the Chinese sample and American sample without splitting. The criteria of ΔCFI no larger in magnitude than − 0.01 and ΔRMSEA less than or equal to 0.015 were adopted as reasonable evidence of invariance [43].

To test our hypothesis about cultural differences in NFA, the scores of our samples and those of 4 independent European samples reported by Appel et al. [22] were compared using a meta-analysis approach. Effect sizes (Hedges’ g values) and associated 95% confidence intervals were calculated. Responses from the American sample to the 5-point English NFA scale were linearly transformed into 7-point scale values by using the formula y = 3(x-3)/2 to match the scale used in the Chinese sample and Appel et al.’s [22] European samples. Additionally, we compared the correlation between the two latent factors (approach and avoidance) across these samples via t-tests after transforming the distribution of the correlations to a normal distribution using Fisher’s z-transformation. The difference in NFC between the Chinese and American samples was also tested using Hedges’ g value.

To explore whether seniors with a highly prevalent public health problem (i.e., hearing loss) have reasonably high intrinsic motivation, as assumed by public health campaigns, we applied MANOVA to examine the differences in the NFA and NFC scores between Chinese seniors with and without hearing loss. Moreover, correlation analysis was conducted to explore the relationship between individuals’ NFA and NFC and the tendency toward early hearing intervention seeking. These analyses were performed using SW 24 (SPSS/IBM, Chicago, IL).

## Results

### Q1. Psychometric characterization of a Chinese version of the NFA

The short 10-item Chinese scale based on Appel et al. [22] showed a test-retest correlation and internal consistency of *r* = .943, *p* < .001, ICC = .942, *p* < .001, and Cronbach’s α = .829, *F* (9,10,485) = 54.580, *p* < .001. Consistent with Kuang, Shi [26], the Chinese NFC scale had an internal consistency above .850, with a Cronbach’s α of .866, *F* (17,19,805) = 99.992, *p* < .001. The relationship between NFA and NFC was assessed as an index of convergent validity [22]. The correlation between NFA and NFC in the Chinese sample was .221 (*p* < .001), which was comparable with that reported by Appel et al. [22] (*r* = .170, *p* < .001), *Z* = 1.19, *p* = .234.

The confirmatory factor analysis indicated that a two-factor model for the 10-item NFA scale, without cross-loadings, had acceptable fit by our criteria in both the Chinese and American samples (Table 2) but did not fit more stringent criteria recommended by Hu and Bentler [45]. As expected, the ESEM and CFA models with cross-loadings yielded even stronger fit indices that passed these more stringent model fit criteria. Standardized factor loadings for the NFA items are shown in Table 3.

**Table 2:** Goodness-of-fit statistics of using Exploratory Structural Equation Modeling (ESEM), a modified Confirmatory Factor Analysis (CFA) model with cross-loadings and a basic CFA model without cross-loadings for the Appel et al.’s 10-item NFA scale in the 4 subsamples

Model tested		λ^**2**^	df	***p***	CFI	TLI	NFI	RMSEA	AIC	ECVI
**ESEM**	Chinese subsample 1	79.325	22	<.001	0.975	0.950	0.967	0.067	165.325	0.285
	Chinese subsample 2	90.266	22	<.001	0.971	0.940	0.962	0.073	176.266	0.302
	American subsample 1	40.860	22	<.001	0.988	0.969	0.974	0.041	126.86	0.251
	American subsample 2	52.681	22	<.001	0.976	0.940	0.960	0.054	138.681	0.294
**Modified** **CFA**	Chinese subsample 1	19.299	14	<.001	0.997	0.991	0.982	0.028	125.299	0.216
	Chinese subsample 2	26.168	14	<.001	0.989	0.974	0.972	0.045	132.168	0.226
	American subsample 1	15.033	14	<.001	0.999	0.997	0.989	0.013	117.033	0.248
	American subsample 2	24.746	14	<.001	0.994	0.983	0.980	0.032	126.746	0.250
**Basic** **CFA**	Chinese subsample 1	83.327	18	<.001	0.971	0.912	0.965	0.089	187.327	0.323
	Chinese subsample 2	86.597	18	<.001	0.969	0.908	0.964	0.090	190.597	0.326
	American subsample 1	28.122	17	<.001	0.983	0.980	0.982	0.035	124.122	0.245
	American subsample 2	35.795	17	<.001	0.982	0.957	0.973	0.046	131.795	0.279
**Criterion for goodness of fit**					≥ 0.9	≥ 0.9	≥ 0.9	≤ 0.1		

Note: *λ*^*2*^ Normed chi-square, *df* Degree of freedom, *CFI* Comparative fit index, *TLI* Turker and Lewis’s Index of fit, *NFI* Normed fit index, *RMSEA* Root mean square error of approximation, *AIC* Akaike Information Criterion, *ECVI* Expected Cross-Validation Index

**Table 3:** Factor loadings of the short version NFA scale 10 items in the Chinese and American samples along with the factor loadings in a German adult sample from Appel et al.’s [22]

	Approach			Avoidance		
	Appel et al. [22]	Chinese	American	Appel et al. [22]	Chinese	American
NFA_3_Appr	.57^*^	.39^*^	**.11**	−.01		
NFA_4_Appr	.42^*^	.51^*^	.66^*^	−.19		
NFA_6_Appr	.74^*^	.60^*^	.69^*^	.07		
NFA_18_Appr	.59^*^	.57^*^	.73^*^	−.13		
NFA_19_Appr	.69^*^	.52^*^	.35^*^	−.00		
NFA_1	.26			.71^*^	.51^*^	.65^*^
NFA_8	−.22			.57^*^	.52^*^	.83^*^
NFA_9	−.19			.46^*^	.56^*^	.82^*^
NFA_10	−.05			.80^*^	**.12**	.94^*^
NFA_11	.02			.74^*^	.41^*^	.85^*^

* ***p*** **< .05**

As shown in Table 4, the NFA measurement structure with two latent factors was invariant between the Chinese and American samples. When all factor loadings were constrained, multigroup analysis indicated noninvariance. Subsequent model tests comparing M4 and M1, and M4 and M0 in Table 4, identified a single noninvariant item (i.e., NFA_10) responsible for the overall noninvariance. Considering that this item only constitutes 1/10 of the model, we chose to relax this specific metric invariance requirement as proposed in the literature [46, 47] while maintaining the rest of the NFA scale. The results of the scalar equivalence and construct variance equivalence tests demonstrated satisfactory factorial invariance that supported comparing the construct means and the correlation of constructs between samples.

**Table 4:** Invariance tests of the basic CFA model between the Chinese group and American group

Model	Description	λ^**2**^	df	p	CFI	TLI	NFI	RMSEA	AIC	ECVI	Test	ΔCFI	ΔRMSEA
M0	Baseline model, no constraints on model parameters	263.124	26	<.001	0.977	0.941	0.966	0.030	607.124	0.283	–	–	–
M1	All factor loadings constrained to be equal	408.270	35	<.001	0.959	0.922	0.947	0.047	704.270	0.329	M1-M0	−0.018	0.017
M2	“Approach” factor loadings constrained to be equal	281.146	31	<.001	0.971	0.949	0.963	0.032	654.146	0.296	M2-M0	−0.006	0.002
M3	“Avoidance” factor loadings constrained to be equal	367.272	31	<.001	0.962	0.943	0.948	0.042	720.272	0.655	M3-M0	−0.015	0.012
M4	All factor loadings constrained to be equal except item NFA_10	292.361	34	<.001	0.969	0.945	0.951	0.039	679.025	0.309	M4-M0	−0.008	0.009
M5	All factor loadings and the vectors of item intercepts constrained to be equal	335.565	73	<.001	0.953	0.929	0.942	0.049	762.515	0.683	M5-M1	−0.006	0.002
M6	All factor loadings and the variances of constructs constrained to be equal	320.461	41	<.001	0.951	0.931	0.944	0.045	738.461	0.398	M6-M1	−0.008	−0.002
M7	All factor loadings and the factor covariance constrained to be equal	518.586	45	<.001	0.946	0.908	0.939	0.061	822.586	0.703	M7-M1	−0.013	0.014

Criteria for accepting the null hypothesis of invariance (Chen et al [43])

≥ − .010

≤.015

*ΔCFI* Change in CFI, *ΔRMSEA* Change in RMSEA

M0: Testing configural invariance between groups, i.e., H_0_ = Both groups associate the same subsets of items with the same constructs;

M1-M0: Testing construct-level metric invariance between groups, i.e., H_0_ = The strength of the relationships between items and their underlying constructs are the same between groups;

M2-M0: Testing the metric invariance of “Approach” between groups, i.e., H_0_ = The strength of the relationships between 5 approach items and the construct “Approach” are the same between groups;

M3-M0: Testing the metric invariance of “Avoidance” between groups, i.e., H_0_ = The strength of the relationships between 5 avoidance items and the construct “Avoidance” are the same between groups;

M4-M0: Testing the metric invariance of item NFA_10 between groups, i.e., H_0_ = The strength of the relationship between NFA_10 and the construct “Avoidance” is the same between groups;

M5-M1: Testing the scalar equivalence of items between groups, i.e., H_0_ = The values of each item corresponding to the zero value of the underlying constructs are the same between groups;

M6-M1: Testing equivalence of construct variances between groups, i.e., H_0_ = The variances of “Approach” and “Avoidance” are the same between groups;

M7-M1: Testing the equivalence of construct covariance between groups, i.e., H_0_ = The covariance between “Approach” and “Avoidance” is the same between groups;

### Q2. Cultural differences in NFA and NFC

**Fig. 2:**
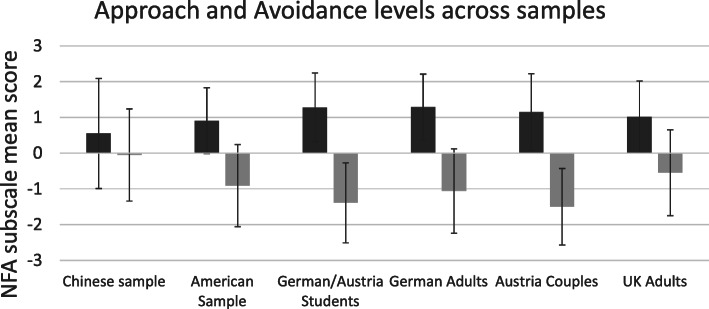
Comparisons of the NFA subscale mean scores across different cultural samples. The r values indicate the correlation between the approach and avoidance scores. * indicates significance at an α level of .05

The mean NFA approach scores and avoidance scores were computed and compared between the Chinese sample and American sample and were also contrasted with the scores of the 4 independent European samples from Appel et al.’s [22] study (see Fig. 2). As shown in Table 5, the effect sizes in terms of the Hedges’ g values and the 95% CIs revealed that the Chinese general public sample had significantly lower NFA approach scores than the American and European samples and significantly higher NFA avoidance scores than people in other cultures. Compared to the European participants, the American adults had significantly lower NFA approach scores and higher avoidance scores. The Chinese general public had significantly lower NFC scores than their American peers.

**Table 5:** The mean, standard deviation, sample size of NFA average subscale scores and NFC total scores in multinational samples, and the effect size indices of the scores comparisons across cultures

	Two samples being compared	Mean ± SD (N)	Cohen’s d	Hedges’ g	SEg	95% CI Lower limit	95% CI Upper limit
Approach	CA vs. AA	.55 ± 1.54 (1186)vs. .91 ± .92 (980)	−0.28	**− 0.28**	0.04	**− 0.36**	**−0.19**
	CA vs. GAS	.55 ± 1.54 (1186)vs. 1.28 ± .96 (1160)	−0.57	**−0.57**	0.04	**−0.65**	**− 0.48**
	CA vs. GA	.55 ± 1.54 (1186)vs. 1.29 ± .92 (627)	−0.54	**−0.54**	0.05	**−0.64**	**− 0.45**
	CA vs. AC	.55 ± 1.54 (1186)vs. 1.15 ± 1.07 (126)	−0.40	**−0.40**	0.09	**−0.58**	**− 0.22**
	CA vs. UKA	.55 ± 1.54 (1186)vs. 1.02 ± 1.0 (236)	−0.32	**−0.32**	0.07	**−0.46**	**− 0.18**
	AA vs. GAS	.91 ± .92 (980) vs. 1.28 ± .96 (1160)	−0.39	**−0.39**	0.04	**−0.48**	**− 0.31**
	AA vs. GA	.91 ± .92 (980) vs. 1.29 ± .92 (627)	−0.41	**−0.41**	0.05	**−0.51**	**− 0.31**
	AA vs. AC	.91 ± .92 (980) vs. 1.15 ± 1.07 (126)	−0.26	**−0.26**	0.09	**−0.44**	**− 0.07**
	AA vs. UKA	.91 ± .92 (980) vs. 1.02 ± 1.0 (236)	−0.12	−0.12	0.07	−0.26	0.02
Avoidance	CA vs. AA	−.05 ± 1.29 (1186) vs. -.91 ± 1.15 (980)	0.70	**0.70**	0.04	**0.61**	**0.79**
	CA vs. GAS	−.05 ± 1.29 (1186) vs. -1.39 ± 1.12 (1160)	1.11	**1.11**	0.04	**1.02**	**1.19**
	CA vs. GA	−.05 ± 1.29 (1186) vs. -1.06 ± 1.18 (627)	0.81	**0.81**	0.05	**0.71**	**0.91**
	CA vs. AC	−.05 ± 1.29 (1186) vs. -1.5 ± 1.07 (126)	1.14	**1.14**	0.10	**0.95**	**1.33**
	CA vs. UKA	−.05 ± 1.29 (1186) vs. -.55 ± 1.2 (236)	0.39	**0.39**	0.07	**0.25**	**0.53**
	AA vs. GAS	−.91 ± 1.15 (980) vs. -1.39 ± 1.12 (1160)	0.42	**0.42**	0.04	**0.34**	**0.51**
	AA vs. GA	−.91 ± 1.15 (980) vs. -1.06 ± 1.18 (627)	0.13	**0.13**	0.05	**0.03**	**0.23**
	AA vs. AC	−.91 ± 1.15 (980) vs. -1.5 ± 1.07 (126)	0.52	**0.52**	0.10	**0.33**	**0.70**
	AA vs. UKA	−.91 ± 1.15 (980) vs. -.55 ± 1.2 (236)	−0.31	**−0.31**	0.07	**−0.45**	**−0.17**
NFC	CA vs. AA	44.90 ± 11.7 (1186) vs. 55.28 ± 11.87 (980)	−0.88	**− 0.88**	0.05	**− 0.97**	**−0.79**

**Note:** *CA* Chinese adults, *AA* American adults, *GAS* German/Austria students, *GA* German adults, *AC* Austria couples, *UKA* UK adults

Sample CA and AA were from the current study, other samples were from Appel et al. [22]

**Fig. 3:**
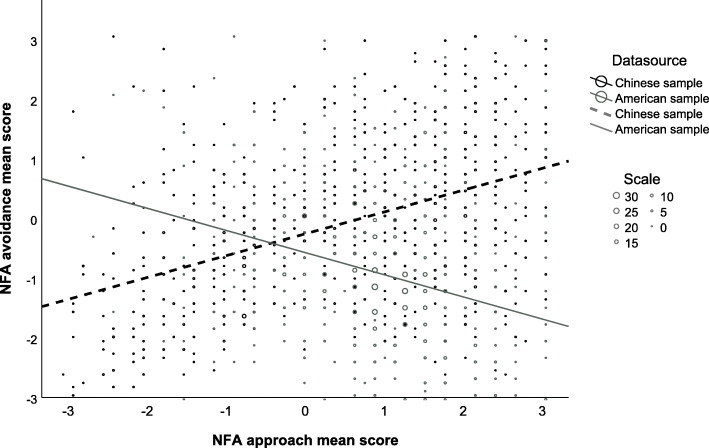
Density plot of the NFA approach and avoidance scores of the Chinese and American samples. The fit lines represent the correlation between the approach and avoidance scores in each sample

Consistent with the finding of the noninvariant construct covariance shown in Table 4 (M7-M1), the correlation between the construct “Approach” and “Avoidance” was different between the Chinese and the American samples. The correlation between the NFA approach and avoidance scores in the Chinese sample was significantly positive, *r* = .44, *p* < .01, in contrast to the negative correlations in the American sample (*r* = −.30, *p* < .01), *Z* = 18.08, *p*<. 001 (see Fig. 3), German/Austrian students (*r* = −.34, *p* < .01), *Z* = 19.79, *p* < .001, German adults (*r* = −.40, *p* < .01), *Z* = 18.11, *p*<. 001, Austrian couples (*r* = −.46, *p* < .01), *Z* = 10.23, *p*<. 001, and UK adults (*r* = −.44, *p* < .01), *Z* = 13.18, *p*<. 001. There was no significant difference in the approach-avoidance correlation between the American sample and the German/Austrian students (*Z* = 1.03, *p*=. 30) or Austrian couples (*Z* = 1.96, *p*=. 05). However, the correlation was significantly smaller in magnitude in the American sample than in the German adults, *Z* = 2.23, *p*=. 030, or the UK adults, *Z* = 2.25, *p*=. 025.

### Q3. Cultural features of NFA and NFC in a population targeted by affective motivation-based public health campaigns

The difference in NFA observed between the Chinese and American general public samples was also present in the older participants (≥60 yrs), with a significantly lower approach score in Chinese seniors (*M* = −.57, *SD* = 1.28) than in American seniors (*M* = .93, *SD* = .81), *F* (1,586) = 167.33, *p* < .001, *η*_p_^2^ = .22, and a significantly higher avoidance score in Chinese seniors (*M* = −.42, *SD* = 1.29) than in American seniors (*M* = −.93, *SD* = 1.09), *F* (1,586) = 16.93, *p* < .001, *η*_p_^2^ = .03.

**Fig. 4:**
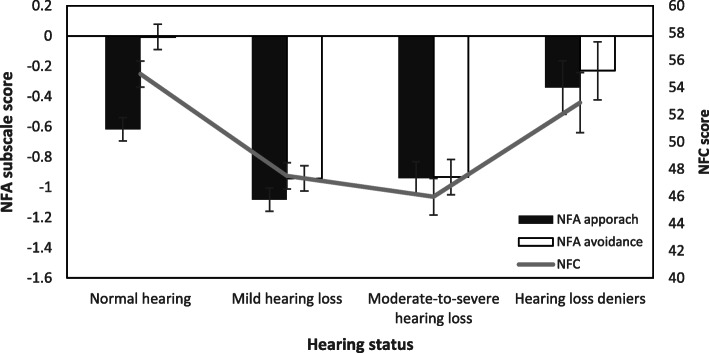
Comparing NFA approach, NFA avoidance and NFC between Chinese seniors with and without hearing loss. Error bars represent ±1 SE

Hearing loss was present in 62.6% of the Chinese seniors in the current study. As shown in Fig. 4, compared to seniors without hearing loss, those who had hearing loss had significantly lower NFA approach scores, *F* (3,482) = 9.00, *p* < .01, *η*_p_^2^ = .05, significantly lower avoidance scores, *F* (3,482) = 26.13, *p* < .01, *η*_p_^2^ = .05, and significantly lower NFC scores, *F* (3,482) = 14.66, *p* < .01, *η*_p_^2^ = .08. There was a small group of individuals (*n* = 34) who reported having normal hearing but failed the pure tone audiometric test and who had similar levels of NFA and NFC (see Table 6) as the normal hearing group.

**Table 6:** The mean and SD of NFA and NFC scores in the Chinese senior participants with different hearing status

	Hearing Status			
	Normal hearing	Mild hearing loss	Moderate-to-severe hearing loss	Hearing loss deniers
NFA approach	−0.62 ± 1.02	−1.08 ± 1.05	− 0.94 ± .88	−0.34 ± 1.31
NFA avoidance	−0.01 ± 1.16	−0.94 ± 1.10	− 0.93 ± 1.01	−0.23 ± 1.27
NFC	54.98 ± 11.94	47.49 ± 13.54	45.98 ± 13.94	52.88 ± 11.60

Intention to avail themselves of early hearing intervention did not differ between groups, *F* (3,482) = 1.01, *p* = .39, *η*_p_^2^ = .01. Regardless of hearing status, participants tended to choose “when I have severe hearing loss and have difficulty communicating with others” in response to the question “When do you think you will consider wearing hearing aids and take serious action?” Of the 486 community-based participants, only 6.8 and 13.8% reported willingness to seek early intervention when they had mild and moderate hearing loss, respectively. Early hearing intervention intention was significantly predicted by NFC, *B* = .174, *t* = 2.662, *p* = .008, but not by the NFA approach score, *B* = −.052, *t* = −.721, *p* = .471, or by the NFA avoidance score, *B* = .025, *t* = .339, *p* = .735.

## Discussion

Considering target audiences’ needs for affect and cognition in contexts involving persuasive communication, such as global public health campaigns, is important because matching messages with receivers’ affective and cognitive orientation can significantly improve the effectiveness of persuasive communication [1]. Cultural differences could be associated with different levels of intrinsic motivation and could pose challenges to public health campaigns aiming to evoke desired attitudes and proactive health-promoting actions. The current study demonstrated cultural differences.

We first developed a Chinese translation of the original English NFA scale [22], which demonstrated acceptable psychometric properties. Based on the results of the ESEM, confirmatory factor analysis and multigroup invariance tests, we found that even a conservative one-level two-factor NFA model was able to had reliability and cross-cultural validity in the Chinese and American samples and can thus be used as a culture-fair assessment of NFA.

Our second question regarded the presence of differences in affective and cognitive intrinsic motivation among the Chinese, American and European samples. The Chinese participants reported lower motivation to approach emotional events and activities and a greater tendency to avoid emotion-inducing events and activities than their American and European counterparts (Fig. 2). This finding is consistent with the literature suggesting that Chinese participants demonstrate more aversion to strong emotions than Americans [48] and value high-arousal affective states less than Americans [13, 14]. Cognitive motivation, or the inclination to engage in and enjoy in-depth thinking and the processing of issue-relevant information [49], was also low in the Chinese sample, as previously observed [17].

A key notion underlying the NFA is that it subsumes both a motivation to approach emotions and a motivation to avoid them [6]. These two motivations are considered somewhat distinct in that approach motivations are closely linked to the experience of positive affect (e.g., gain), while avoidance motivations are closely linked to negative affect (e.g., loss) [50, 51]. That said, NFA approach and avoidance both focus on people’s attitude toward emotion as an end in itself, i.e., is emotion (positive and negative) something they want to approach or avoid? [22] In this way, they are different from emotion regulation. Behavior is regulated by these two distinct motivations, according to theories of individual differences in motivation [52–54]. People with a predominant approach orientation are more responsive to cues of reward, while people with a predominant avoidance orientation are more responsive to cues of threat and punishment [53]. Hence, it is valuable to examine emotion approach and emotion avoidance separately.

In the present study, whereas NFA approach and avoidance were negatively correlated in the American and European samples, they were positively correlated in the Chinese sample. This counterintuitive motivation to approach emotions might be explained by observations regarding differential reactions to emotions (e.g., strategies to regulate emotions) across cultures [55]. According to Maio [6], people do not approach emotions if they regard them as unproductive or uncomfortable. In other words, if emotions are not generally appreciated by a society, people may prefer to keep them to themselves; their intention to approach emotions may thus be low or, if it is high, may be accompanied by a high level of restraint. Cultures that have a long-term orientation, that have embeddedness values, and that are hierarchical (e.g., East Asian cultures) tend to have higher scores on emotion suppression, and the correlation between emotion reappraisal and suppression tends to be positive, whereas Western cultures demonstrate an inverse relationship between affective approach and avoidance. Potentially, Chinese people have higher emotional ambivalence [56], where emotion desire is often compromised by concerns about the consequences of emotional expression and by efforts to refrain from emotional experience and expression. The goal of these exchanges is to avoid interpersonal conflict and maintain harmony [57], given that Chinese culture places strong emphasis on a harmonious and balanced relationship with nature and in social interaction [58]. As a result, affect-based motivation in Chinese culture may vary along a mixed emotion-coping continuum from low approach and low avoidance to high approach and high avoidance (rather than from high approach to high avoidance), with a precondition of maintaining balance and harmony in interpersonal and social relationships, which is not typical in Western cultures. We did not assess other affective constructs, personality traits, or culture-representative characteristics (e.g., individualism vs. collectivism, analytic vs. holistic, independent vs. interdependent) due to our lack of a priori model involving these constructs. The current study’s focus was to illustrate overall differences in affective/cognitive intrinsic motivation in the Chinese public compared to American and European samples. Thus, mechanisms of the observed cultural differences in NFA remain an open question for future research.

The low correlations between NFC and NFA within the samples (~.2) but the reliable sample-related differences could also suggest that cultural factors are responsible for differences in NFC. When people value harmonious social relationships, they tend to put less effort into information search and deliberation and into the utilization of issue-relevant information to think, reason and form attitudes and behaviors different from the mainstream [18]. These social strategies reflect low cognitive intrinsic motivation and might be a barrier to persuasive communication [59]. Indeed, as the NFC is essentially an affective scale assessing how enjoyable cognition is to an individual, given that Chinese generally were only 50% certain about whether approaching/avoiding emotions was enjoyable or not (Fig. 2), they might benefit less from emotions and therefore may be less likely to approach cognition than people in other cultures.

Our third objective was to explore whether the intrinsic motivation level of community-based seniors in China with a specific highly prevalent public health condition (i.e., hearing loss) is high, which would warrant affectively motivated health campaigns. Given the reduction in NFA and NFC in Chinese seniors relative to American seniors, Chinese seniors with hearing loss showed even lower NFA and NFC than those without hearing loss. This result differed from our hypothesis that NFA and NFC would be similar in seniors with and without hearing loss. The particularly low level of NFA in seniors with hearing loss, might be associated with the link between hearing loss and late-life depression [60], the latter typically indicating motivation disturbance [61]. The decreased level of NFC might be explained by Spotts [62], who reported that age-related declines in cognitive ability could affect NFC. Hearing loss is known to be associated with cognitive decline in older adults [63]; therefore, their cognitive motivations might be constrained by their cognitive capabilities.

Early hearing intervention intention was low regardless of hearing status; the majority of older Chinese people preferred to wait until their hearing condition became difficult to cope with. Our community-based geriatric sample was representative of the urban older population targeted by hearing health campaigns in China. It had a hearing loss prevalence of 62.6%, which is consistent with a previous report [64]. Low levels of NFA and NFC could help explain why geriatric hearing health campaigns in China are less successful than those in America, although Chinese campaigns have followed the American model [20]. A growing body of research in the U.S. has emphasized the importance of affective appeals in efficiently delivering health promotion messages [2]. However, perceptions of affective benefits (e.g., reduced anxiety, depression and stress; elevated self-confidence) and/or instrumental/cognitive outcomes (e.g., reduced risk of developing severe tinnitus and dementia) require a matching level of affective and/or cognitive intrinsic motivation in intended audiences. Our data suggest that this assumption should be questioned.

As the attempt to establish health goals by affective means has a relatively short history in China, the lack of a relationship between NFA (approach or avoidance) and intention to engage in early hearing intervention was not surprising. Given the numerous cultural differences associated with emotion [65], exploring strategies in which messaging is matched to a culture’s need for affect could be helpful in cross-cultural health campaigns. The presence of an association between NFC and early hearing intervention indicates that more effective persuasion might be possible in the Chinese geriatric population if hearing health campaign messages are framed to more specifically reflect the cognitive benefits that audiences actually value to match their low NFC. For example, early intervention could make you feel less “different” from your community (this is important for Chinese individuals). Alternatively, if a subdimension of NFC, such as NFC approach or NFC avoidance, as derived in other translations of the NFC scale [66], is found to be differentially relevant to health intentions, messages could be tailored to match that specific NFC subdimension to increase their persuasive power. We recommend research on these perspectives.

Our study has multiple limitations. First, limited demographic information was collected from the samples. Although the Chinese and American samples were comparable in terms of sex distribution (*χ*^2^(1) = 1.782, *p* = .182), only 55% of the age data were available in the American sample,. Other demographic differences (e.g., education, SES), as well as variables associated in the literature with affective style (e.g., neurodevelopmental history, disease exposure history, social network richness, frontal EEG asymmetry, and exposure to weather phenomena) [67–69], might moderate our results. However, public health campaigns normally target audiences at the population level rather than the individual level, and as such, it would have been inappropriate to analyze individual differences in the current study. Our large-scale public samples were representative of the campaign target audiences who have access to the campaign messages usually spread on social media in both countries and advocated across community sites in China, so examining cross-cultural differences in NFA and NFC in those samples, as a whole, is more appropriate for our purposes. Individual differences were not controlled for or covaried, as this would require full analysis and understanding of potential moderation effects [70], which is beyond the scope of the current work. Second, as the original [22] scale scored items from 1 to 7 and the American version scored items from 1 to 5, we could not be consistent with both; our version inherited its scale from Appel’s [22] sample and was thus inconsistent with the American version in terms of scoring. We addressed this inconsistency by linearly converting responses from the American dataset to a 7-point scale, which could have distorted the NFA mean values of the American sample and biased the results. However, this conversion caused only a 0.17% change in the mean value and was unlikely to account for the 6% difference between the means on the 7-point scale. For future research reference, we reported the Chinese and American NFA norms by age and sex on both the 5- and 7-point scales (see supplementary material). The third limitation is the lack of hearing assessment and early hearing intervention intention data from the American sample. As a result, we were unable to directly compare the NFA and NFC levels of seniors with hearing loss between the two cultures. In addition, without data on how individuals in any of our samples responded to an actual public health campaign, all our considerations regarding campaigns, at this point, remain speculation; future research is necessary to confirm them.

These limitations notwithstanding, our results demonstrate a lack of affective and cognitive intrinsic motivation in Chinese individuals compared with the American public. These differences point to a potential challenge in framing effective messages for some cultures. Ideally, recognizing and understanding this challenge will inspire the consideration of novel persuasive strategies for these audiences. For example, using our example of public campaigns targeting hearing loss, instead of replicating the Western hearing healthcare campaign model, messages could target Asian values more specifically, such as by emphasizing a person’s ability to be in harmony with nature (e.g., hearing waterfalls) and society (e.g., reducing others’ communication burden and stress) rather than individual affective and cognitive goals. Another direction might be strengthening the influence of messages using newly available technology. Breves and Heber [71] reported that people low in NFA were influenced by immersive media, while people high in NFA were not, potentially because sensory-rich media experiences offer greater assistance to individuals with lower trait predispositions [72].

## Conclusions

We conclude that the need for affect (NFA) construct may transcend its Western origins. Assessing NFA and NFC could inform global public health campaigns by helping them frame messages consistent with audiences’ culture-dependent intrinsic motivation.

## Supplementary Information

**Additional file 1: Appendix** The Chinese version of the Need for Affect-Short Scale. The Chinese translation of the 10-item NFA scale.

**Additional file 2: Supplement** Normative NFA values of the general Chinese and American public. The Chinese and American norms of Need for Affect approach and avoidance subscale scores by age and gender in 7-point and 5-point rating scales.

## Data Availability

The datasets used and/or analyzed during the current study are available from the corresponding author on reasonable request.

## Abbreviations

NFC: Need for Cognition
NFA: Need for Affect
MANOVA: Multivariate analysis of variance
ESEM: Exploratory Structural Equation Modeling
FIML: Information Maximum Likelihood
CFI: Comparative fit index
NFI: Normed fit index
RMSEA: Root mean square error of approximation

## References

[1] Haddock G, Maio GR, Arnold K, Huskinson T (2008). Should persuasion be affective or cognitive? The moderating effects of need for affect and need for cognition. Personal Soc Psychol Bull.

[2] Conner M, Rhodes RE, Morris B, Mceachan R, Lawton R (2011). Changing exercise through targeting affective or cognitive attitudes. Psychol Health.

[3] Strauss K, Parker SK, O'Shea D (2017). When does proactivity have a cost? Motivation at work moderates the effects of proactive work behavior on employee job strain. J Vocat Behav.

[4] Aquino A, Alparone FR, Pagliaro S, Haddock G, Maio GR, Perrucci MG (2020). Sense or sensibility? The neuro-functional basis of the structural matching effect in persuasion. Cogn Affect Behav Neurosci.

[5] Teeny JD, Siev JJ, Briol P, Petty RE. A review and conceptual framework for understanding personalized matching effects in persuasion. J Consum Psychol. 2020. 10.1002/jcpy.1198.

[6] Maio GR, Esses VM (2001). The need for affect: individual differences in the motivation to approach or avoid emotions. J Pers.

[7] Cacioppo JT, Petty RE (1982). The need for cognition. J Pers Soc Psychol.

[8] Curşeu PL (2006). Need for cognition and rationality in decision-making. Stud Psychol (Bratisl).

[9] Sandra DA, Otto AR (2018). Cognitive capacity limitations and need for cognition differentially predict reward-induced cognitive effort expenditure. Cognition..

[10] Gellert P, Ziegelmann JP, Schwarzer R (2012). Affective and health-related outcome expectancies for physical activity in older adults. Psychol Health.

[11] Lim N (2016). Cultural differences in emotion: differences in emotional arousal level between the east and the West. Integr Med Res.

[12] Oyserman D, Coon HM, Kemmelmeier M (2002). Rethinking individualism and collectivism: evaluation of theoretical assumptions and meta-analyses. Psychol Bull.

[13] Tsai JL, Brian K, Fung HH (2006). Cultural variation in affect valuation. J Pers Soc Psychol.

[14] Sims T, Tsai JL, Jiang D, Wang Y, Fung HH, Zhang X (2015). Wanting to maximize the positive and minimize the negative: implications for mixed affective experience in American and Chinese contexts. J Pers Soc Psychol.

[15] Bontempo RN, Bottom WP, Weber EU (1997). Cross-cultural differences in risk perception: a model-based approach. Risk Anal.

[16] Lam KC, Buehler R, Mcfarland C, Ross M, Cheung I (2005). Cultural differences in affective forecasting: the role of focalism. Personal Soc Psychol Bull.

[17] Sanders J, Gass R, Wiseman R, Bruschke J (1992). Ethnic comparison and measurement of argumentativeness, verbal aggressiveness, and need for cognition. Commun Rep.

[18] Chan S (1999). The Chinese learner-a question of style. Educ Train.

[19] Goman AM, Lin FR (2016). Prevalence of hearing loss by severity in the United States. Am J Public Health.

[20] Zhao F, Manchaiah V, Claire LS, Danermark B, Jones L, Brandreth M (2015). Exploring the influence of culture on hearing help-seeking and hearing-aid uptake. Int J Audiol.

[21] Chien W, Lin FR (2012). Prevalence of hearing aid use among older adults in the United States. Arch Intern Med.

[22] Appel M, Gnambs T, Maio GR (2012). A short measure of the need for affect. J Pers Assess.

[23] Arceneaux K, Vander Wielen RJ (2013). The effects of need for cognition and need for affect on partisan evaluations. Polit Psychol.

[24] Widhiarso W. A note on emotion words translation on different cultures. SSRN Electron J. 2009. 10.2139/ssrn.1505307.

[25] Barger B, Nabi R, Hong LY (2010). Standard back-translation procedures may not capture proper emotion concepts: a case study of Chinese disgust terms. Emotion..

[26] Kuang Y, Shi JQ, Cai YQ (2005). The Chinese version of need for cognition scale. Chin Ment Health J.

[27] Zhang M, Bi ZR, Fu XP, Wang JF, Ruan QW, Zhao C (2019). A parsimonious approach for screening moderate-to-profound hearing loss in a community-dwelling geriatric population based on a decision tree analysis. BMC Genet.

[28] Wang G, Li C, Guan W, Xiong J, Kuang S, Hu Y. Development and evaluation of reliability and validity of the Chinese version of HHIE-S. J Audiol Speech Pathol. 2014;22(6):568–72.

[29] Fokkema M, Greiff S. How performing PCA and CFA on the same data equals trouble. Hogrefe Publishing; 2017.

[30] Tabachnick BG, Fidell LS, Ullman JB (2007). Using multivariate statistics.

[31] Greiff S, Heene M. Why psychological assessment needs to start worrying about model fit. Hogrefe Publishing; 2017.

[32] Maydeu-Olivares A. Assessing the size of model misfit in structural equation models. Psychometrika. 2017;82(3):533–58. 10.1007/s11336-016-9552-7 28176040

[33] McNeish D, An J, Hancock GR (2018). The thorny relation between measurement quality and fit index cutoffs in latent variable models. J Pers Assess.

[34] Bentler PM, Bonett DG (1980). Significance tests and goodness of fit in the analysis of covariance structures. Psychol Bull.

[35] Bentler PM (1990). Comparative fit indexes in structural models. Psychol Bull.

[36] Chen F, Curran PJ, Bollen KA, Kirby J, Paxton P (2008). An empirical evaluation of the use of fixed cutoff points in RMSEA test statistic in structural equation models. Sociol Methods Res.

[37] Young JQ, John M, Thakker K, Friedman K, Sugarman R, Sewell JL (2021). Evidence for validity for the cognitive load inventory for handoffs. Med Educ.

[38] Thanh ND, Quyen BT, Tien TQ (2016). Validation of a brief CES-D scale for measuring depression and its associated predictors among adolescents in chi Linh, Hai Duong, Vietnam. AIMS Public Health.

[39] Karimi FZ, Alesheikh A, Pakravan S, Abdollahi M, Damough M, Anbaran ZK (2017). Surveying the factor structure and reliability of the Persian version of the Jefferson scale of physician lifelong learning (JeffSPLL) in staff of medical sciences. Electron Physician.

[40] Cohen J, Cohen P, West SG, Aiken LS. Applied multiple regression/correlation analysis for the behavioral sciences. Routledge; 2013.

[41] Yu L, Shek DTL (2014). Testing factorial invariance across groups: an illustration using AMOS. Int J Disabil Hum Dev.

[42] Cheung GW, Rensvold RB (2002). Evaluating goodness-of-fit indexes for testing measurement invariance. Struct Equation Model Multidiscipl J.

[43] Chen FF (2007). Sensitivity of goodness of fit indexes to lack of measurement invariance. Struct Equ Model Multidiscip J.

[44] Rutkowski L, Svetina D (2014). Assessing the hypothesis of measurement invariance in the context of large-scale international surveys. Educ Psychol Meas.

[45] Hu LT, Bentler PM (1995). Evaluating model fit. In: RH IH, editor. Structural equation modeling: concepts, issues, and applications.

[46] Byrne BM, Shavelson RJ, Muthén B (1989). Testing for the equivalence of factor covariance and mean structures: the issue of partial measurement invariance. Psychol Bull.

[47] Marsh HW, Hocevar D (1985). Application of confirmatory factor analysis to the study of self-concept: first- and higher order factor models and their invariance across groups. Psychol Bull.

[48] Eid M, Diener E (2001). Norms for experiencing emotions in different cultures: inter- and Intranational differences. J Pers Soc Psychol.

[49] Petty RE, Briñol P, Loersch C, McCaslin MJ, Leary MR, Hoyle RH (2009). The need for cognition. Handbook of individual differences in social behavior.

[50] Sherman DK, Mann T, Updegraff JA (2006). Approach/avoidance motivation, message framing, and health behavior: understanding the congruency effect. Motiv Emot.

[51] Davidson RJ (1998). Affective style and affective disorders: perspectives from affective neuroscience. Cognit Emot.

[52] Tversky A, Kahneman D (1981). The framing of decisions and the psychology of choice. Science..

[53] Carver CS, Sutton SK, Scheier MF (2000). Action, emotion, and personality: emerging conceptual integration. Personal Soc Psychol Bull.

[54] Gray JA (1990). Brain systems that mediate both emotion and cognition. Cognit Emot.

[55] Matsumoto D, Yoo SH, Nakagawa S (2008). Culture, emotion regulation, and adjustment. J Pers Soc Psychol.

[56] Chen SX, Cheung FM, Bond MH, Leung JP (2005). Decomposing the construct of ambivalence over emotional expression in a Chinese cultural context. Eur J Personal.

[57] Cheung FM, Leung K, Zhang J-X, Sun H-F, Gan Y-Q, Song W-Z (2001). Indigenous Chinese personality constructs: is the five-factor model complete?. J Cross-Cult Psychol.

[58] Wang L, Juslin H (2009). The impact of Chinese culture on corporate social responsibility: the harmony approach. J Bus Ethics.

[59] Lassiter GD, Briggs MA, Bowman RE (1991). Need for cognition and the perception of ongoing behavior. Personal Soc Psychol Bull.

[60] Rutherford BR, Brewster K, Golub JS, Kim AH, Roose SP. Sensation and psychiatry: linking age-related hearing loss to late-life depression and cognitive decline. Am J Psychiatry. 2018;175(3):215–24. 10.1176/appi.ajp.2017.17040423 PMC5849471 29202654

[61] Forsell Y, Jorm AF, Winblad B. Association of age, sex, cognitive dysfunction, and disability with major depressive symptoms in an elderly sample. Am J Psychiatry. 1994;151(11):1600–4. 10.1176/ajp.151.11.1600 7943447

[62] Spotts H. Evidence of a relationship between need for cognition and chronological age: implications for persuasion in consumer research. In: Allen CT, John DR, editors. NA - Advances in Consumer Research Volume 21. Provo: Association for Consumer Research; 1994. p. 238–243.

[63] Lin FR, Yaffe K, Xia J, Xue Q-L, Harris TB, Purchase-Helzner E (2013). Hearing loss and cognitive decline in older adults. JAMA Intern Med.

[64] Wang Y, Mo L, Li Y, Zheng Z, Yu Q (2016). Analysing use of the Chinese HHIE-S for hearing screening of elderly in a northeastern industrial area of China. Int Audiol.

[65] Tsai EH-L (2005). A cross-cultural study of the influence of perceived positive outcomes on participation in regular active recreation: Hong Kong and Australian university students. Leis Sci.

[66] Aquino A, Laura P, Alparone FR (2018). Validation of the Italian version of the need for cognition scale-short version. BPA Appl Psychol Bull.

[67] Wheeler RE, Davidson RJ, Tomarken AJ (2010). Frontal brain asymmetry and emotional reactivity: a biological substrate of affective style. Psychophysiology..

[68] Tottenham N (2014). The importance of early experiences for neuro-affective development. Curr Top Behav Neurosci.

[69] Beecher ME, Eggett D, Erekson D, Rees LB, Bingham J, Klundt J (2016). Sunshine on my shoulders: weather, pollution, and emotional distress. J Affect Disord.

[70] Miller GA, Chapman JP (2001). Misunderstanding analysis of covariance. J Abnorm Psychol.

[71] Breves P, Heber V (2020). Into the wild: the effects of 360° immersive nature videos on feelings of commitment to the environment. Environ Commun.

[72] Ahn SJ, Bostick J, Ogle E, Nowak KL, McGillicuddy KT, Bailenson JN (2016). Experiencing nature: embodying animals in immersive virtual environments increases inclusion of nature in self and involvement with nature. J Comput-Mediat Commun.

