# Supplementary material for: Are need for affect and cognition culture dependent? Implications for global public health campaigns: a cross-sectional study
Source: BMC Public Health. 2021 Apr 9;21:693. doi: 10.1186/s12889-021-10689-w (PMC8034077; doi:10.1186/s12889-021-10689-w)
Supplement: Supplementary file 1 — Additional file 1: Appendix The Chinese version of the Need for Affect-Short Scale. The Chinese translation of the 10-item NFA scale. [file 12889_2021_10689_MOESM1_ESM.docx]

**Appendix**

The Need for Affect-Short Scale (Chinese version)

情感需求量表中文简化版

Please indicate how much you agree with the following statements about you using the following 7-point scale from –3 (strongly disagree) to 3 (strongly agree).

请选择下列陈述是否准确地描述了您。

| **-3** | **-2** | **-1** | **0** | **1** | **2** | **3** |
| --- | --- | --- | --- | --- | --- | --- |
| Strongly disagree  当然不是 | Disagree  不是 | Somewhat disagree  不太对 | Uncertain  我不确定 | Somewhat agree  有点对 | Agree  是的 | Strongly agree  的确是这样 |

| Score  得分 | # | Item  问卷项 |
| --- | --- | --- |
| . | 3* | I feel that I need to experience strong emotions regularly.  我感觉每隔一阵子就需要激动一下。 |
|  | 4* | Emotions help people get along in life.  各种情绪有助于人们生活顺利。 |
| . | 6* | I think that it is important to explore my feelings.  我觉得深入了解自己的感受很重要。 |
| . | 18* | It is important for me to be in touch with my feelings.  对我来说，体会自己的感受很重要。 |
| . | 19* | It is important for me to know how others are feeling.  了解别人的感受对我来说很重要。 |
| . | 1 | If I reflect on my past, I see that I tend to be afraid of feeling emotions.  过去，我内心有些害怕去感受自己的情绪**。** |
| . | 8 | I find strong emotions overwhelming and therefore try to avoid them.  我发现情绪激动时就像排山倒海，势不可挡，所以我尽量避免。 |
| . | 9 | I would prefer not to experience either the lows or highs of emotion.  我既不想情绪低落，也不想情绪高涨。 |
| . | 10 | I do not know how to handle my emotions, so I avoid them.  我不知道如何处理好自己的情绪，所以我选择逃避。 |
| . | 11 | Emotions are dangerous—they tend to get me into situations that I would rather avoid.  我觉得情绪是个危险的东西，容易使我陷入我不愿面对的处境。 |
| *Items in the Approach subscale | | |
