# Supplementary material for: Are need for affect and cognition culture dependent? Implications for global public health campaigns: a cross-sectional study
Source: BMC Public Health. 2021 Apr 9;21:693. doi: 10.1186/s12889-021-10689-w (PMC8034077; doi:10.1186/s12889-021-10689-w)
Supplement: Supplementary file 2 — Additional file 2: Supplement Normative NFA values of the general Chinese and American public. The Chinese and American norms of Need for Affect approach and avoidance subscale scores by age and gender in 7-point and 5-point rating scales. [file 12889_2021_10689_MOESM2_ESM.docx]

**Supplement**

**26-item NFA scale vs. 10-item NFA scale**

A basic one-level two-factor (approach and avoidance) model of NFA which constrained cross-loadings and covariance among items to be zero was first tested via confirmatory factor analysis. The indices of the 26-item scale suggested a poor fit of this model to the current data (CFI=.692, TLI=.664, NFI=.664, RMSEA=.109 in the Chinese sample, and CFI=.842, TLI=.814, NFI=.790, RMSEA=.067 in the American sample), while the indices of the 10-item suggested an insufficient fit but comparable between the Chinese and American samples (CFI=.852, TLI=.810, NFI=.840, RMSEA=.130 in the Chinese sample, and CFI=.885, TLI=.820, NFI=.867, RMSEA=.100 in the American sample). For a culture-fair NFA assessment purpose, the Appel et al.’s ^1^ 10-item scale was used for further model testing.

Table 1. The Chinese and American norms of Need for Affect approach and avoidance subscale scores by age and gender in 7-point and 5-point rating scales. Note that the age information was missing for 45% of the American sample.

|  |  |  |  | **7-point Likert scale (-3~3)** | | **5-point Likert scale (1~5)** | |
| --- | --- | --- | --- | --- | --- | --- | --- |
|  | Gender | Age | n | Approach M(SD) | Avoidance M(SD) | Approach M(SD) | Avoidance M(SD) |
| **American** | All |  | 980 | .95(.89) | -.88(1.18) | 3.61(.61) | 2.40(.77) |
|  | male | 18-25 | 6 | 1.60(.68) | -.55(1.35) | 4.07(.45) | 2.63(.90) |
|  |  | 26-35 | 21 | .87(.75) | -.64(1.22) | 3.58(.50) | 2.57(.82) |
|  |  | 36-45 | 23 | .77(.80) | -.53(1.16) | 3.51(.54) | 2.64(.77) |
|  |  | 46-55 | 40 | 1.00(.94) | -.88(1.23) | 3.67(.62) | 2.42(.82) |
|  |  | 56-65 | 41 | .65(.84) | -.90(1.05) | 3.44(.56) | 2.40(.70) |
|  |  | 66-75 | 14 | .71(.67) | -.79(.79) | 3.48(.45) | 2.47(.52) |
|  |  | >75 | 0 | -- | -- | -- | -- |
|  |  | Total | 145 | .84(.84) | -.77(1.13) | 3.47(.65) | 2.49(.76) |
|  | female | 18-25 | 18 | .78(1.10) | -.57(1.23) | 3.52(.74) | 2.62(.82) |
|  |  | 26-35 | 55 | 1.04(1.04) | -.66(1.30) | 3.69(.69) | 2.56(.87) |
|  |  | 36-45 | 74 | .84(.88) | -.88(1.16) | 3.56(.59) | 2.41(.77) |
|  |  | 46-55 | 114 | 1.03(.93) | -.1.03(1.24) | 3.68(.61) | 2.31(.83) |
|  |  | 56-65 | 90 | 1.03(.77) | -.96(1.14) | 3.69(.52) | 2.36(.76) |
|  |  | 66-75 | 31 | 1.03(.83) | -1.09(1.02) | 3.68(.55) | 2.27(.68) |
|  |  | >75 | 12 | 1.32(1.05) | -1.00(1.33) | 3.88(.70) | 2.24(.89) |
|  |  | Total | 394 | .99(.91) | -.92(1.20) | 3.67(.59) | 2.35(.77) |
| **Chinese** | All |  | 1166 | .55(1.54) | -0.05(1.29) | 3.37(1.03) | 2.97(.86) |
|  | male | 18-25 | 113 | 1.69(.87) | .24(1.33) | 4.13(.58) | 3.16(.89) |
|  |  | 26-35 | 74 | 1.66(.91) | .58(1.19) | 4.11(.61) | 3.38(.80) |
|  |  | 36-45 | 20 | .67(1.55) | .29(1.47) | 3.45(1.03) | 3.19(.98) |
|  |  | 46-55 | 24 | -.40(1.47) | .07(1.00) | 2.73(.98) | 3.04(.67) |
|  |  | 56-65 | 50 | -.23(1.32) | -.02(1.30) | 2.85(.88) | 2.99(.86) |
|  |  | 66-75 | 77 | -.78(1.15) | -.57(1.30) | 2.48(.77) | 2.62(.87) |
|  |  | >75 | 47 | -.56(1.35) | -.30(1.39) | 2.63(.90) | 2.80(.93) |
|  |  | Total | 405 | .55(1.53) | -.06(1.34) | 3.36(1.05) | 3.03(.90) |
|  | female | 18-25 | 269 | 1.73(.78) | .15(1.23) | 4.16(.52) | 3.10(.82) |
|  |  | 26-35 | 88 | 1.38(1.07) | .14(1.18) | 3.92(.71) | 3.10(.79) |
|  |  | 36-45 | 33 | .36(1.45) | -.02(1.16) | 3.24(.97) | 2.99(.77) |
|  |  | 46-55 | 47 | .16(1.27) | .31(1.17) | 3.11(.85) | 3.21(.78) |
|  |  | 56-65 | 138 | -.23(1.34) | -.14(1.25) | 2.85(.89) | 2.90(.84) |
|  |  | 66-75 | 142 | -.68(1.17) | -.62(1.26) | 2.54(.78) | 2.59(.84) |
|  |  | >75 | 44 | -1.18(1.09) | -.87(1.02) | 2.21(.73) | 2.42(.68) |
|  |  | Total | 761 | .56(1.53) | -.10(1.26) | 3.37(1.02) | 2.93(.84) |

**References**

1. Appel M, Gnambs T and Maio GR. A short measure of the need for affect. *J Pers Assess* 2012; 94: 418-426.
